# Implementation of totally robotic right hemicolectomy: lessons learned from a prospective cohort

**DOI:** 10.1007/s11701-023-01646-3

**Published:** 2023-06-21

**Authors:** Jeremy Meyer, Jan Wijsman, Rogier Crolla, George van der Schelling

**Affiliations:** 1 Department of Surgery, Amphia Hospital, Molengracht 21, 4811GX Breda, The Netherlands; 2 Division of Digestive Surgery, University Hospitals of Geneva, Rue Gabrielle-Perret-Gentil 4, 1211 Geneva 14, Switzerland; 3 Medical School, University of Geneva, Rue Michel-Servet 1, 1206 Geneva, Switzerland

**Keywords:** Robotic surgery, MIS, RHC, Colorectal cancer, Colon cancer

## Abstract

Robotics facilitates the realization of intra-corporeal anastomosis during right hemicolectomy and allows extracting the operative specimen through a C-section, offering potential benefits in terms of post-operative recovery and incidence of incisional hernia. Therefore, we progressively implemented robotic right hemicolectomy (robRHC) in our centre, and would like to report our initial experience with the technique. Consecutive patients who underwent robRHC within a single centre were prospectively included. Variables related to patients’ demographics, surgical procedures, post-operative recovery and pathological outcomes were collected. Sixty patients underwent robRHC in our centre. Indications for robRHC were colon cancer in 58 patients (96.7%) and polyps not amenable to endoscopic resection in 2 patients (3.3%). Fifty-eight patients underwent robRHC with D2 lymphadenectomy and central vessel ligation (96.7%), and two patients (3.3%) had robRHC associated with another procedure. All patients had intra-corporeal anastomosis. The mean ± operative time was of 200.4 ± 114.9 min. Two conversions (3.3%) to open surgery were performed. The mean ± SD length of stay was of 5.4 ± 3.8 days. Seven patients (11.7%) experienced a post-operative complication with a Clavien–Dindo score ≥ 2. Two patients (3.5%) had an anastomotic leak. The mean ± SD number of harvested lymph nodes was of 22.4 ± 7.6. All patients had negative pathological margins (R0 resection). To conclude, robotic RHC is a safe procedure, which can be implemented with satisfying peri- and post-operative outcomes. The potential benefits of the technique remain to be demonstrated by randomized controlled trials.

**Supplementary Information:**

The online version contains supplementary material available at 10.1007/s11701-023-01646-3.

## Introduction

Professional societies recommend choosing a minimally invasive approach over open surgery for performing right hemicolectomy (RHC) due to its improved peri-operative and post-operative outcomes with similar long-term oncological outcomes [1–6].

Laparoscopic RHC (lapRHC) is the most frequent approach for performing minimally RHC, and robotic RHC (robRHC) is only offered in a small proportion of patients undergoing RHC [7–9]. Because of the technical limitations of laparoscopy, most centres adopted extra-corporeal anastomosis [7] and midline incision as the extraction site [10–12, 14] as the standard of care during lapRHC. However, intra-corporeal anastomosis [10, 13–16] and off-midline extraction sites [10, 17] are associated with better post-operative outcomes. Considering that robotics provides finer tissue dissection and facilitates realization of intra-corporeal anastomosis [7, 18], robotics could potentially improve the outcomes of minimally invasive RHC when compared to lapRHC.

In our centre, we started performing robRHC with intra-corporeal anastomosis and would like to report our initial experience with the technique.

## Methods

### Type of study

The study is a monocentric prospective cohort study, and was performed according to the STROBE guideline (Table S1).

### Population

Consecutive patients who underwent robRHC during years 2014–2022 were included in a prospective database. No exclusion was performed.

### Surgical procedure

Robotic RHC was performed using the Da Vinci Si (Intuitive Surgical, Sunnyvale, USA) from 2014 to August 2019, and the Da Vinci Xi (Intuitive Surgical, Sunnyvale, USA) from September 2019 onwards.

The following surgical procedure is described for the Da Vinci Xi.

**Fig. 1:**
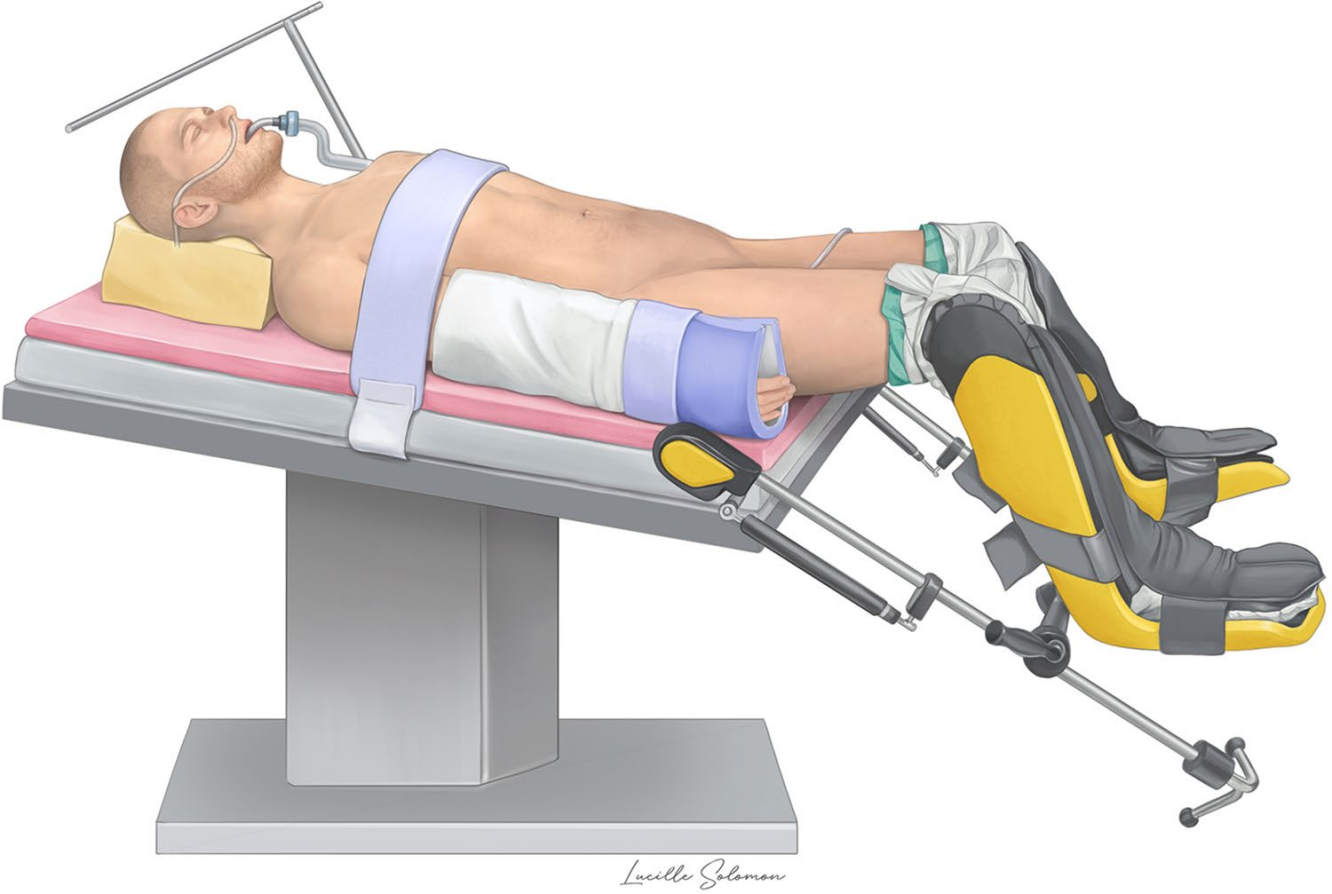
Patient positioning

Selective bowel decontamination (Tobramycine and Colistine) was administered before surgery [19]. The patient was placed in modified Lloyd-Davies position, with slight reverse Trendelenburg, on a commercial anti-sliding mat, with the arms tucked alongside the body, and the legs bent with the thighs flat (Fig. 1). Disinfection and draping were done as usual. Pneumoperitoneum was insufflated with a Veress needle at the Palmer’s point or, alternatively, with an open approach between future robotic ports 2 and 3.

**Fig. 2:**
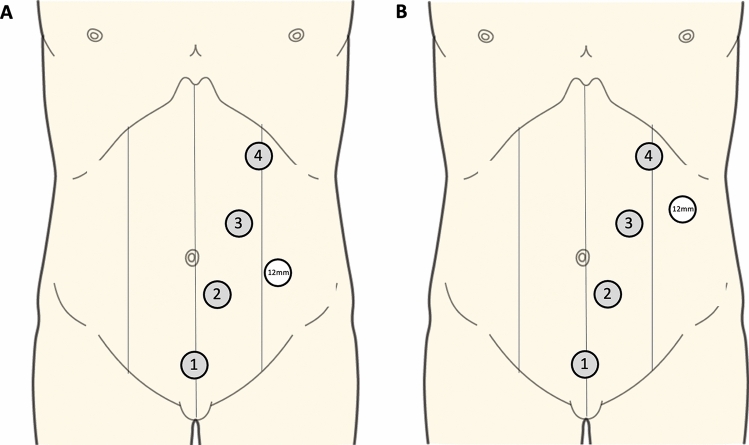
Ports placement for performing robotic right hemicolectomy using the Da Vinci Xi. The robotic camera is inserted in port number 2, Fenestrated bipolar forceps are introduced in port number 1, monopolar scissors in port number 3 and the small grasping retractor in port number 4. A 12 mm assistant port is placed between ports 2 and 3 (**A**) or between ports 3 and 4 (**B**)

When the pneumoperitoneum was insufflated, bony prominences were marked. An 8 cm wide horizontal line was traced at two fingertips above the pubis as future site of C-section. Thereafter, a line was drawn starting at the middle of the future C-section to the midclavicular line at 2 fingertips from the left subcostal rim. Future positions of robotic ports were indicated at equidistance on this line. If not done previously, a 12 mm Optiview port was inserted under visual control in the left flank between future ports 2 and 3 (Fig. 2). Alternatively, this port could be inserted between ports 3 and 4. Eight millimetres robotic ports were inserted on the sites previously marked. Exploration of the abdomen was performed using the robotic camera. Eventual adhesions were taken down using laparoscopic instruments, and localization of the cancer/polyp was verified by checking the tattoo made during pre-operative endoscopy (excepted for caecal tumours/polyps). The table was given 10° of reverse Trendelenburg and 15° of left tilt. The small bowel was put into the left lower quadrant, and the omentum was put into the upper abdomen. The Da Vinci XI surgical robot was brought from the right side of the patient and docked. A fenestrated bipolar forceps was inserted in port n°1, the robotic camera in port n°2, monopolar scissors in port n°3 and the small grasping retractor in port n°4. A medial to lateral mobilization of the right colon was performed. Alternatively, a bottom-up approach could be done. Briefly, the ileo-colic was put into tension by grasping the appendix or the ileocolic mesentery with arm n°4 and pulling it ventrally into the right iliac fossa.

**Fig. 3:**
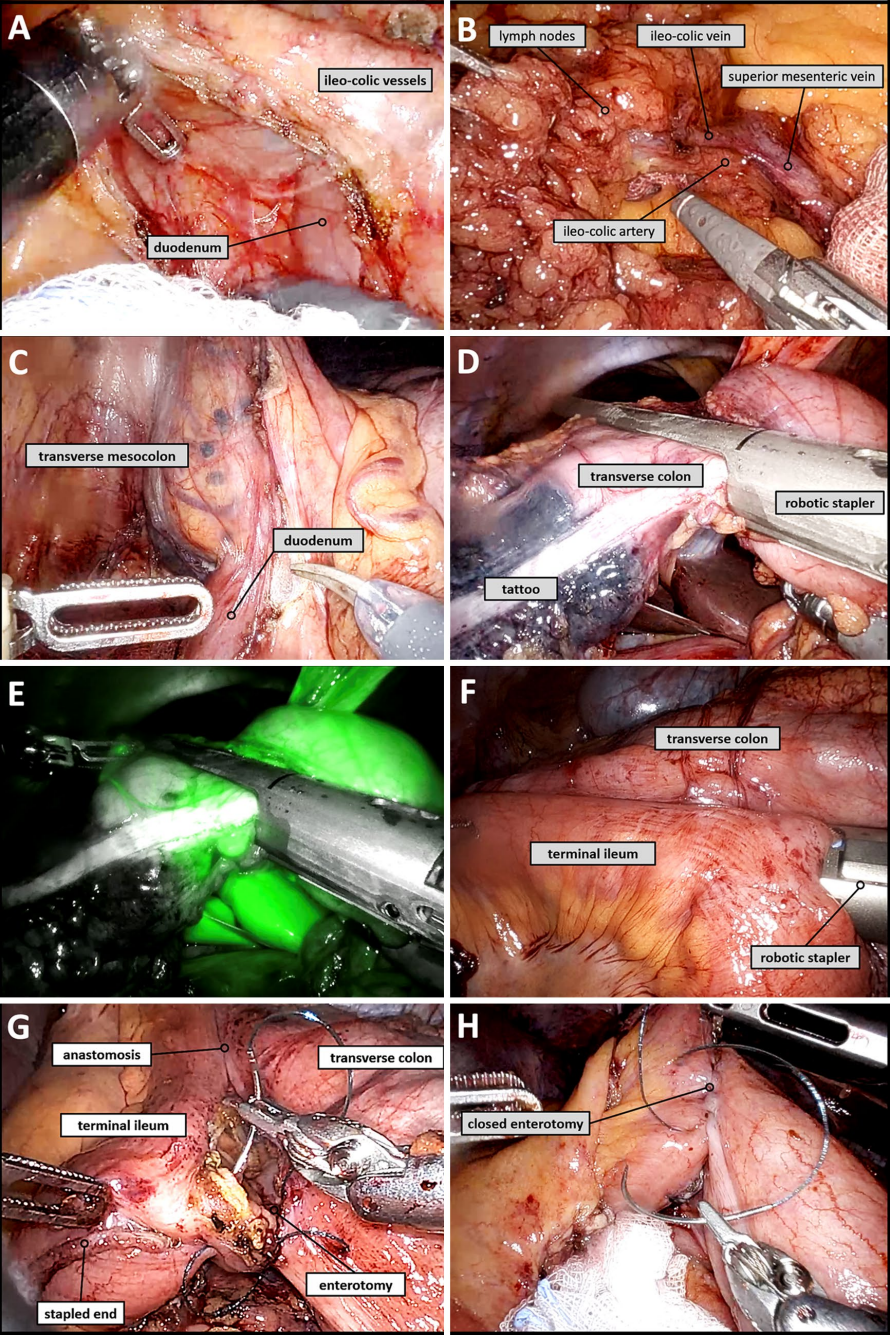
Steps for robotic right hemicolectomy. **A** Exposure of the origin of the ileo-colic vessels and of the duodenum. **B** Lymphadenectomy and central vascular ligation performed at the origin of the ileo-colic vessels. **C** Separation of the transverse mesocolon from the retroperitoneal plane and the duodenum. **D** Division of the transverse mesocolon using the robotic vessel sealer, and of the transverse colon using the robotic stapler (the tattoo was at a safe distance from the distal margin of the tumour). **E** ICG fluorescence angiography of the transverse colon. **F** Stapled side-to-side ileo-colic anastomosis using the robotic stapler. **G** and **H** Closure of the enterotomy using a V-lock

Once the ileo-colic pedicle was identified, the peritoneum was incised below it and the plane was developed until identifying the duodenum (Fig. 3A). Thereafter, the ileo-colic vessels were isolated at their origins (central vascular ligation) (Fig. 3B). The plane was further developed, dissecting laterally over Gerota’s fascia (sometimes underneath Gerota’s fascia carefully sparing the gonadal vessels and ureter if the tumour was adherent to Gerota’s fascia) separating the transverse mesocolon from the retroperitoneum and the duodenum (Fig. 3C). The anterior surface of the pancreatic head was exposed, and all the mesocolon located on the right side of the superior mesenteric vein was removed up to the pancreatic head. Once identified, the eventual right colic vessels and the right branches of the middle colic vessels were controlled at their origins close to the superior mesenteric vein (central vascular ligation, not shown). Therefore, lymph node stations 201, 211, 221, 202 and 212 were removed. Removal of lymph node station 222 depended on the localization of the tumour (hepatic flexure or not). The small bowel mesentery and the transverse mesocolon were divided using the vessel sealer (introduced in port n°3) or with a bipolar forceps and hemolock clips (not shown).

The 8 mm port n°3 or, alternatively port n°4, was replaced by a 12 mm port. The small bowel and the transverse colon (Fig. 3D and E) were divided using the Sureform robotic stapler after ICG perfusion angiography (built-in Firefly system). The small bowel and the transverse colon were placed side-by-side in an isoperistaltic fashion, and a side-to-side anastomosis was performed using the Sureform robotic stapler (Fig. 3F). The enterotomy was then closed using a V-lock (Fig. 3G and H). The specimen was extracted through a C-section protected with a wound retractor. Post-operative follow-up was performed according to an enhanced rehabilitation after surgery protocol.

### Variables of interest

Gender, age at time of surgery, ASA score, date of surgery, operative time, type of surgical procedure performed, type of robotic system used, conversion to open surgery, TNM stage of the operative specimen, incidence and Clavien–Dindo grade of post-operative complication, as well as length of stay, were extracted from the prospective database.

## Results

In our centre, 60 patients underwent robRHC (Fig. 1). Twenty-eight patients (46.7%) were males. The mean ± SD age was 72.8 ± 8.9 years. Three patients (5%) were considered as ASA 1, 35 patients (58.3%) as ASA 2, 19 patients (31.7%) as ASA 3 and 3 patients (5%) as ASA 4. Indications for robRHC were colon cancer in 58 patients (96.7%) and polyps not accessible to endoscopic resection in 2 patients (3.3%) (Table 1).

**Table 1:** Baseline patients’ characteristics

	Patients (*n = *60)
Gender (male), *n* (%)	28 (46.7%)
Age, mean (SD)	72.8 (8.9)
ASA score, *n* (%)	
- ASA 1	3 (5%)
ASA 2	35 (58.3%)
ASA 3	19 (31.7%)
ASA 4	3 (5%)
Surgical indication, *n* (%)	
Colon cancer	58 (96.7%)
Polyp	2 (3.3%)

RobRHC was performed using the Da Vinci Si (Intuitive Surgicals, Sunnyvale, USA) in 35 patients (58.3%), and using the Da Vinci Xi (Intuitive Surgicals, Sunnyvale, USA) in 25 patients (41.7%). Fifty-eight patients underwent robRHC only (96.7%), and two patients (3.3%) had robRHC associated with another procedure: one patient (1.7%) had robRHC with synchronous liver metastasis resection and one patient (1.7%) had robRHC with synchronous adrenal gland resection.

The mean ± operative time was of 200.4 ± 114.9 min. Two conversions (3.3%) to open surgery were performed. Reasons for conversion to open surgery were a bulky tumour in one patient, and doubts regarding the vascular anatomy in the second patient. In the other patients, right colon mobilization, vessel division and lymphadenectomy, as well as ileo-colic anastomosis were performed intra-corporeally.

**Fig. 4:**
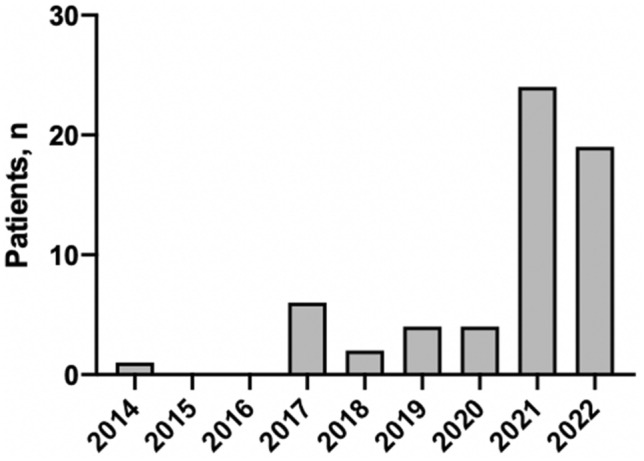
Number of robRHC procedures performed per year

The mean ± SD length of stay was of 5.4 ± 3.8 days. Seven patients (11.7%) experienced a post-operative complication with a Clavien–Dindo score ≥ 2. Two patients (3.5%) had an anastomotic leak (Table 2, Fig. 4). The first anastomotic leak occurred on post-operative day 3 and the anastomotic defect could be secondarily closed by laparotomy, allowing to preserve the anastomosis. The patient recovered well with drainage and antibiotics, and could be discharged on post-operative day 12. The second anastomotic leak occurred on post-operative day 4 and was managed by laparoscopy, also with secondarily closure, drainage and antibiotics. The patient was discharged on post-operative day 11.

**Table 2:** Intraoperative and perioperative outcomes of robRHC in our centre

	Patients (*n = *60)
Operative time, min	
Range	110–900
Mean (SD)	200.4 (114.9)
Median	180
Conversion to open surgery, *n* (%)	2 (3.3%)
Incidence of post-operative complication^a^	7 (11.7%)
Incidence of anastomotic leak, *n* (%)	2 (3.5%)
Length of postoperative stay, day	
Range	2–25
Mean (SD)	5.4 (3.8)
Median	4

^a^Clavien–Dindo ≥ 2

From a pathological point of view, the mean ± SD number of harvested lymph nodes was of 22.4 ± 7.6. All patients had negative resection margins (R0). The definitive TNM stage was TNM I in 8 patients (17.8%), TNM II in 17 patients (37.8%), TNM III in 16 patients (35.6%) and TNM 4 in 4 patients (8.9%).

## Discussion

Robotics in colorectal surgery was initially reserved for rectal cancer surgery in patients in whom laparoscopy would have shown technical limitations [20–22]. However, with increasing access to the robotic platforms, indications for robotic surgery expanded in colorectal surgery. Noteworthy, the improved suturing capacities offered by the robotic platforms are getting valorised in procedures, such as rectopexy and RHC. In our cohort, this translated into a dramatic increase of the numbers of robRHC over the recent years. Furthermore, robRHC is considered as an ideal training procedure for surgeons experienced in laparoscopic surgery who wish to make the transition to robotic surgery [23], which helps the procedure to gain in popularity.

Initial reports from other teams documented a mean operative time of 327.5 min for performing robRHC [24] and was slightly shorter (285.6 ± 71.5 min) in a cohort of a single experienced surgeon who performed 108 robRHC [23]. In our cohort of patients, the mean operative time (defined as the skin-to-skin time) was of 200.4 ± 114.9 min, probably because the three consultant surgeons performing robRHC in our centre were already experienced in robotic colorectal surgery and had cumulated a significant number of (low) anterior resections for rectal cancer [20–22]. Therefore, we believe that operative time during robRHC, which is longer than for lapRHC [25], could be reduced with experience in robotic surgery. On this aspect, the ideal number of robRHC procedures to reduce operative time and conversion to open surgery was reported to be of 44 cases per surgeon [23]. Still, it will probably never match the shorter operative times of lapRHC, but we feel that economics is not the most important outcome measure, particularly from a patient’s point of view.

In experienced hands, robRHC seems to be a safe procedure. For instance, in our cohort, only 3.3% of the robRHC procedures had to be converted to open surgery and the incidence of post-operative complications was of 11.7%. On this aspect, a systematic review and meta-analysis of comparative studies demonstrated that robRHC had a significantly smaller incidence of conversion to open surgery (OR 0.34, 95% CI 0.15–0.75) and a smaller incidence of post-operative complication than lapRHC (OR 0.73, 95% CI 0.52–1.01) [26].

However, due to the lack of high-quality randomized evidence showing the potential benefits of robRHC over lapRHC, as well as the increased cost of the technique [27], current recommendations fail to strongly support rRHC [28]. To our knowledge, only one randomized controlled trial compared robRHC with lapRHC, and did not show any difference between the two techniques in terms of length of stay, complications, postoperative pain, number of harvested lymph nodes [18], as well as 5-year disease-free and overall survivals [27]. However, in this trial, surgeons could choose the type of anastomosis to perform (intra-corporeal or extra-corporeal). Therefore, one of the advantages of the robotic approach, which is the improved suturing ability (and facilitation of intra-corporeal anastomosis [7, 18]), could not be properly evaluated. Moreover, powering of the sample size was made on the length of stay, which is modulated by numerous factors and institutional guidelines and may not be optimal to assess the eventual superiority of the technique. In our opinion, the combination of a stable camera, tri-dimensional vision and stabilized movements of the instruments allows a much safer approach to the vascular dissection than in laparoscopy. It also allows more careful assessment and dissection when encountering T4 invasion. We believe that recovery of the bowel function may constitute a better primary outcome, as robotics allows finer dissection of the tissues, facilitates the realization of intra-corporeal (which is associated with better post-operative outcomes [10, 13–16]) and was shown by meta-analyses of observational studies to shorten the time to flatus and first stools [25, 29, 30]. Moreover, robotics facilitates the realization of minimally invasive complete mesocolic excision for those who perform the technique.

The robotic approach also has some downsides when performing RHC. For instance, the limited operational space related to the positioning of the robotic arms (when using the Da Vinci Xi) may give rise to technical difficulties, notably in elongated right colons. To counter this limitation, it is important to always finish dissecting the planes all the way to the end, in order to avoid leaving in place some attachments which may change the anatomic positioning of the colon. We also recommend to give careful attention to the patient’s positioning (as illustrated in Fig. 1) in order to anticipate and avoid potential mechanical conflicts with the robotic arms. This is particularly true if positioning of the robotic ports differs from our recommendation (Fig. 2) and adopts the sub-umbilical linear configuration (as used for performing complete mesocolic excision, for example).

To conclude, we believe that robotics could potentially contribute to generalize a fully minimally invasive surgery approach for performing RHC, including realization of intra-corporeal anastomosis, which could translate into better post-operative recovery outcomes when compared to the current standard approach, which is lapRHC with extra-corporeal anastomosis. So far, in experienced hands, the technique is safe. However, superiority of the robRHC over lapRHC in terms of short-term recovery outcomes remains to be demonstrated by randomized controlled trials.

## Supplementary Information

Below is the link to the electronic supplementary material.

Supplementary file1 (DOCX 29 KB)

## Data Availability

Data is available upon reasonable request.

## Abbreviations

lapRHC: Laparoscopic right hemicolectomy
RHC: Right hemicolectomy
robRHC: Robotic right hemicolectomy
